# Habitat Heterogeneity and Channelization of Small Rivers and Streams: Fish Community Responses to Ecological Gradients

**DOI:** 10.1002/ece3.72092

**Published:** 2025-09-05

**Authors:** Xiaohao Shi, Robert Czerniawski

**Affiliations:** ^1^ Department of Hydrobiology, Institute of Biology University of Szczecin Szczecin Poland

**Keywords:** community assembly, conservation, disturbance, diversity, Drawa catchment

## Abstract

Physical habitat gradients in small rivers and streams profoundly influence aquatic community structure. These ecosystems are critical for biodiversity conservation, serving as refugia and nurseries for numerous species. Effective freshwater conservation necessitates tailored strategies addressing specific anthropogenic pressures and each habitat type's unique geomorphological and hydrological characteristics. This study examined the response of fish communities to habitat heterogeneity and channelization in small rivers and streams within the Drawa River catchment, Pomerania, northwestern Poland. The interaction of morphohydrochemical characteristics created distinct spatial patterns within the catchment. Critical physical habitat characteristics significantly influencing fish community structure were identified through Canonical Correspondence Analysis (CCA). Aquatic macrophyte coverage showed a negative relationship with both fish biomass and abundance in the catchment. Habitat diversity, mediated by physical factors and their interactions, significantly influences the fish community in the catchment. More abundant species have a greater impact on beta diversity patterns, while sites with fewer fish often have unique fish communities. This study underscores the importance of prioritizing rivers with simplified substrates and anthropogenic modifications for conservation and restoration efforts. Furthermore, sites lacking fish populations warrant consideration in conservation planning. Optimal reconstruction strategies to mitigate anthropogenic impacts may include enhancing substrate complexity, removing obstructions, constructing fish passages, and reconfiguring channels. In‐stream habitat improvement emerges as the primary strategy for protecting side channels in temperate lowland catchments.

## Graphical abstract

Side channels are critical for fish habitat conservation in fluvial systems. Physical habitat gradients structure fish assemblies. Regulation, simplified substrate, and dense vegetation cover negatively impact fish communities in rivers and streams, highlighting the importance of habitat diversity and channel restoration for effective fish conservation.

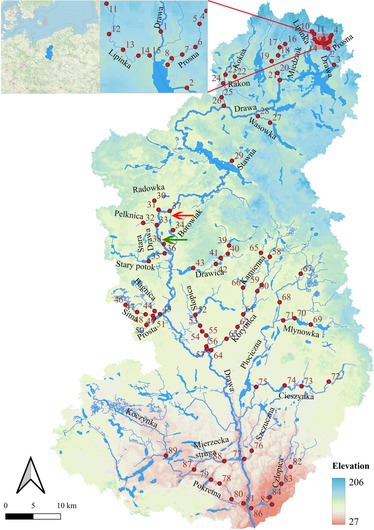

## 1 Introduction

Freshwater ecosystems, harboring 40% of all fish species, are experiencing an alarming acceleration in extinction rates (Watson et al. 2021). Unlike their terrestrial or marine counterparts, freshwater species have a lower chance of expanding their habitats due to several unique characteristics, such as fragmented, linear, and downstream‐flowing water systems (Arthington et al. 2016). Consequently, the need for conservation and restoration efforts for freshwater ecosystems is increasing globally, particularly in protecting endangered species and preserving species‐rich habitats (Arthington et al. 2016).

The streams in a catchment and its side channels are vital for maintaining the life cycles of many species. Most fishes complete their life cycles within the confines of a single river segment, except for amphidromous species. This is facilitated by the complex and diverse habitats formed by various geomorphic and hydraulic units (Wolter et al. 2016). Small rivers and streams are excellent spawning and nursery areas, fulfilling the nursery requirements for young fish populations, which require special environmental conditions such as shallow, slow flow, and diverse substrates (Yager et al. 2013; Stoffers et al. 2022). Furthermore, as crucial refugia, side channels provide favorable environmental conditions for growth. These conditions include diminished water velocity, moderated temperature fluctuations, and augmented food availability (Bellmore et al. 2013; Collas et al. 2019). However, small rivers and streams are susceptible to environmental changes like climate and flow, and are vulnerable to human intervention, supporting low biodiversity individually but high regional diversity (Fieseler and Wolter 2006). Thus, the habitat protection of small rivers is crucial for aquatic ecosystem conservation and effective management.

Environmental gradient acts as the primary driver in shaping the composition of ecological communities (Giam and Olden 2016). There is a pivotal influence of habitat gradients on the composition of freshwater fish assemblages across a range of spatial scales, concluding that environmental heterogeneity generates distribution patterns in the assemblages of fishes along habitat gradients (Szalóky et al. 2021). Environmental gradients shape stream fish assemblages through multiple interacting factors—biological, chemical, topographic, and physical in‐stream variables—which operate at different scales linked to geological conditions (Maasri et al. 2021). Habitat gradients are fundamental to understanding ecosystem function, as demonstrated by several key aspects. Substrate type influences microhabitat use and spawning behavior, while riverbank characteristics affect refuge availability, with both factors influencing flow velocity and community composition (Pander and Geist 2010; Hubbell and Banford 2019). Stream size relates to energy flow and riparian cover, while river morphology features such as bends and meanders create flow refugia and hydraulic dead zones that support species richness (Finlay 2011; Garcia et al. 2012). Fish communities respond to different levels of disturbance, which can be used to indicate environmental pressure and understand community structure and ecosystem health across habitat gradients (Xu et al. 2021; Di Lorenzo et al. 2022).

Worldwide, there are more than 800,000 dams, and over 70% of large rivers in Europe, North America, and the former Soviet Union are heavily regulated, affecting approximately two thirds of freshwater flow (Petrić et al. 2019). The impacts of regulation through river channelization vary due to different dam and barrier types, sizes, and local conditions, creating distinct site‐specific challenges and varying restoration potential. Even minor channelization of lowland meandering rivers can alter natural morphology, particularly affecting sandy substrate distribution, while high degrees of channelization result in freshwater species decline (Graf et al. 2016; Dutta et al. 2018). Effective conservation strategies for diverse habitats require tailoring approaches to address specific human pressures and unique geomorphological and hydrological characteristics. To achieve this goal, comprehensive ichthyofaunal data is crucial for effective conservation and restoration efforts.

Existing studies on the fish community of small rivers and streams within the Drawa River basin primarily focused on individual species distribution and migration, neglecting community assessments (Bartel 1987; Chelkowski et al. 1996; Fredrich et al. 2008; Dębowski et al. 2016). Therefore, we assessed the fish community in the catchment of the Drawa River as an example to understand how spatial patterns vary across different environmental gradients, including areas influenced by human activities and natural processes. Physical habitat gradients are crucial factors shaping the distribution of diversity and community structures, and we predict that in‐stream habitat heterogeneity and channelization will be particularly crucial physical environmental conditions for the fish community within the catchment. To elucidate the status of fish communities in the catchment, we assessed (1) the characterization of habitat gradients across sites based on environmental variables, using multivariate analysis (PCA, Principal Component Analysis); (2) how fish community composition and diversity vary along these gradients, using ordination (CCA, Canonical Correspondence Analysis), Hill numbers, and beta‐diversity; and (3) the variation in the internal structure of the communities based on biomass–abundance relationships to explore shifts in dominance patterns across habitat types. The study provides a detailed insight into how fish communities and species respond to habitat heterogeneity and channelization in small rivers and streams. Results offer a scientific foundation for protecting and conserving side channels in temperate regions.

## 2 Methods

### 2.1 Study Area and Fish Survey Protocols

**FIGURE 1:**
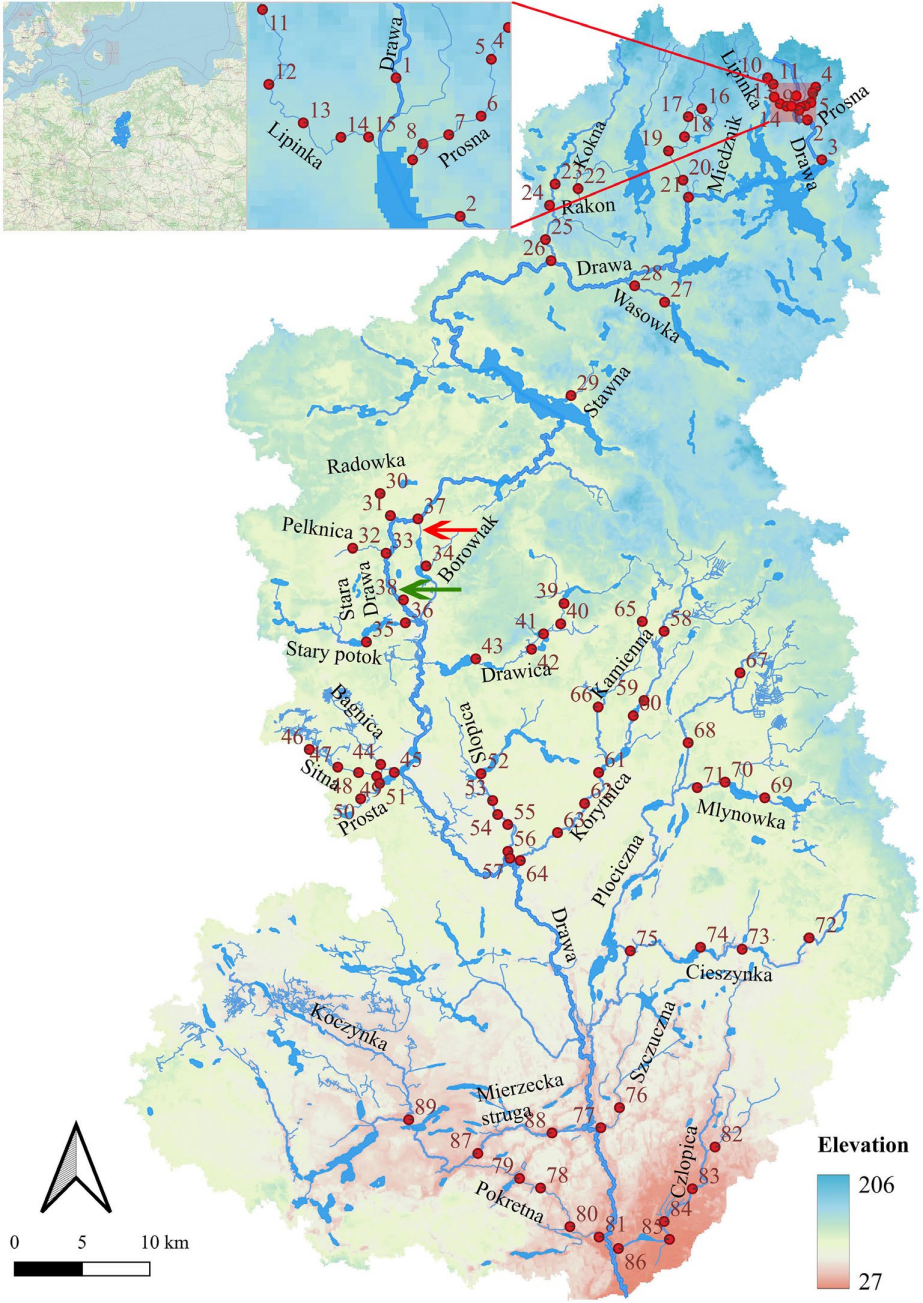
Locations of 89 fish sampling sites within the Drawa River catchment, Pomerania (summer 2010). The red arrow indicates the artificially straightened section of the main channel, while the green arrow marks the original, natural course of the Drawa River.

A field study was conducted in the summer of 2010 within the catchment of the Drawa River, Pomerania, northwestern Poland (Figure 1), as delineated using QGIS software (QGIS Development Team 2016). The Drawa, with a catchment area reaching 3289 km^2^, has a total length of 185.9 km. It discharges into the lower Noteć River, constituting a quaternary tributary of the Oder River system (Kubiak‐Wójcicka and Kornaś 2015). It encompasses three natural reserve areas and provides habitat for a diverse assemblage of threatened species, such as European eel *Anguilla anguilla* (L. 1758), Burbot *Lota lota* (L. 1758), Common nase *Chondrostoma nasus* (L. 1758), and Bullhead *Cottus gobio* (L. 1758). The river is a typical mid‐sized temperate lowland river with an altitude range of 27–206 m. The river flows through broad plains, and its bottom is mainly composed of sand, gravel, and stones, offering a diversified habitat. The rivers and streams studied in this research varied in width from 0.7 to 8 m and depth from 0.05 to 6 m. Their discharge ranged from 0.0025 to 0.914 m^3^/s, and their current velocity varied from 0.008 to 0.7 m/s during the study period. The catchment is predominantly an agricultural‐forestry area, with forests and semi‐natural ecosystems covering 59.9% of the total area (Kubiak‐Wójcicka and Kornaś 2015). Over the past century, the hydromorphological characteristics of the Drawa catchment have undergone extensive artificial transformations, primarily due to the demands of agricultural irrigation and wood transportation. The most significant modification occurred in the middle reach of Stara Drawa, where the highly meandering channel (shown as a green line in Figure 1) was replaced with a straightened channel (shown as a red line in Figure 1), completely altering the river's main course. Notably, numerous channels were built and channelized in the headwater and sub‐channels, with most located in the middle and lower reaches of the catchment, as shown in Figure 1.

Fish sampling was conducted at 89 sites that belong to the headwater section of the Drawa River and its 27 tributary branches in the catchment. Sites 1–29 are located in the upper reach, sites 30–66 in the middle reach, and sites 67–89 in the lower reach of the catchment. The fish sampling was conducted using electric equipment (IG200 Hans Grassl, Germany). At each station, a 200‐m stretch was sampled, with equipment settings adjusted according to local conditions. Sampling locations were typically spaced 1–8 km apart. Sites located less than 1 km apart were chosen based on either physical barriers (small hydropower installations, culverts, flood gates, road crossings, or beaver dams) or distinct differences in stream environments. All individuals were identified at the species level, and their total length (precise to within 0.1 cm) and total weight (precise to within 0.1 g) were measured. All the fish were anesthetized in a 2% concentration of MS‐222 (Tricaine). After measurements, fish were immediately released into the river to minimize stress and ensure survival.

### 2.2 Physical–Chemical Habitat Conditions

#### 2.2.1 Water Hydrological Conditions

Water hydrological conditions were assessed at 89 sites using an electromagnetic water flow sensor (OTT Hydromet, Germany) to measure water current velocity (m/s), riverbed width (m), and water depth (m). Total phosphorus (mg/L) and total nitrogen (mg/L) were quantified using a Hach Lange DR 890 photometer (Hach Lange, Loveland, CO, USA). The following water quality parameters were measured using a Hydrolab DS5 multiprobe (Hydrolab, Loveland, CO, USA): temperature (°C), dissolved oxygen (mg/L), pH, conductivity (S/m), chlorophyll a (mg/L), chloride (mg/L), total dissolved solids (TDS) (mg/L), ammonia (mg/L), nitrite (mg/L), nitrate (mg/L), and suspended solids (mg/L). All measurements were conducted during midday hours.

#### 2.2.2 Physical Gradients

Protocols for classifying physical habitats should consider ecological, practical, and geomorphologically insightful factors (Thomson et al. 2001). Small rivers and streams were grouped based on readily identifiable environmental heterogeneity using a hierarchical approach that considered physical conditions and human intervention levels (Figure S1 in Data S1).

1. Sampling sites were categorized based on the level of artificial intervention: natural (18 sites) retaining their natural topography, semi‐natural (34 sites) with some human‐induced morphological changes but preserving natural features, and regulated (37 sites) heavily modified for transportation, hydropower, irrigation, or flood control.
2. Sampling sites were categorized based on dominant substrate type: (a) sand (56 sites), (b) sand with mixed gravel (19 sites), and (c) mixed sand, gravel, and stones (14 sites).
3. River courses were categorized based on sinuosity: straight (30 sites), winding (50 sites), and very winding (9 sites).
4. Aquatic macrophytes (plants living submerged, floating, or emerging from the water; Chambers et al. 2008), as the dominant plants in freshwater ecosystems, shape the environment by modifying light, temperature, water flow, and bottom structure, making them an essential engineer of aquatic habitats (Carpenter and Lodge 1986). Consequently, it is regarded as a physical factor in this study. Macrophyte coverage was assessed by estimating the percentage of macrophyte cover at the river bottom of each sampling site.

Physical habitat characteristics for each site are listed in Table S1.

### 2.3 Diversity Indices

To assess how fish biodiversity varies across physical habitat gradients, we used Hill numbers to quantify species diversity (Jost 2006). Hill numbers, also known as effective numbers of species, provide a unified framework for measuring α diversity. These diversity indices form a unified framework that effectively incorporates both species richness and relative abundance. The parameter *q* determines the indices' sensitivity to species relative abundances, allowing for a comprehensive assessment of community structure (Ellison 2010; Chao et al. 2014). Hill numbers describe diversity at different scales: q0 (Species richness) counts the total number of species present, q1 (Exponential of Shannon index) represents the number of effectively common species, and q2 (Inverse Simpson index) indicates the number of effectively dominant species in a community.

To determine beta diversity patterns in the catchment, we analyzed two key metrics: Species Contributions to Beta Diversity (SCBD) and Local Contributions to Beta Diversity (LCBD) (Legendre and De Cáceres 2013). SCBD measures how individual species influence overall beta diversity patterns across a region. Species with high SCBD values often exhibit distinctive characteristics such as distinct distribution patterns, abundance variation across sites, specific niche requirements, or limited spatial ranges. Meanwhile, LCBD indicates how unique each sampling site's community composition is compared to other sites in the study area, and these values can be correlated with environmental variables to understand what drives site uniqueness. This analytical framework has become a valuable tool for conservation planning (da Silva et al. 2018; Xia et al. 2022).

### 2.4 Data Analysis

Eight‐nine sampling sites were surveyed, but seven (4, 6, 10–12, 20, and 82) lacked fish and were excluded from further community analyses. R software version 4.3.2 was used for all statistical analyses (R Core Team 2013). Graphical illustrations were created using the ggplot2 package and QGIS software (QGIS Development Team 2016; Wickham 2016).

#### 2.4.1 Habitat Gradient

Prior to analysis, numerical environmental variables were log (*x* + 1) transformed to reduce skewness and standardized for analysis. Categorical variables were converted to binary (dummy) variables. We then assessed multicollinearity between environmental variables using Variance Inflation Factors (VIF), excluding those with VIF values exceeding 4 to prevent redundant information in subsequent analyses (Garcia et al. 2015). Nine numerical variables were retained: water temperature, dissolved oxygen, pH, total nitrogen, total phosphorus, suspended solids, current velocity, depth, and macrophyte cover. Categorical variables included substrate type, river intervention status, and river course, from which six dummy variables were selected: sand and sand/gravel, regulated and semi‐natural, and winding and very winding. PCA was employed to explore patterns of environmental variation and habitat heterogeneity. Subsequently, the k‐means clustering algorithm (with *k* = 3) was applied to the PCA scores to identify potential natural groupings of sites based on their overall habitat conditions. Prior to PCA, all variables were standardized to *Z*‐scores. Multicollinearity among the environmental variables was evaluated using the vif function from the usdm package, and PCA was conducted using the prcomp function within the stats package (Babak et al. 2014).

Using site abundance data, we first applied Detrended Correspondence Analysis (DCA) to investigate prevailing species response patterns along environmental gradients. A first‐axis gradient length of 4.30 SD units (> 4) indicated high beta diversity and full species turnover (Hill and Gauch 1980; Holland 2008). Based on this result, we selected CCA to examine the relationships between fish species composition and environmental variables, and to identify the key environmental drivers shaping community structure in the catchment (ter Braak and Verdonschot 1995). The raw site abundance data were used for the CCA analysis and were standardized using a Chi‐square transformation as part of the analysis process. The significance of the CCA model and individual environmental variables was tested using permutation tests (999 permutations). DCA and CCA were performed using the vegan package (decorana and cca function), with multicollinearity among environmental variables assessed using the usdm package (vif function; Oksanen et al. 2015).

#### 2.4.2 Diversity Indices

##### 2.4.2.1 α Diversity

The hillR package was used to calculate Hill numbers using the hill_taxa function (Li 2018). To assess how Hill diversity indices (q0, q1, and q2) respond to key habitat variables—river intervention status, substrate type, and macrophyte cover—identified as significant in the CCA, we applied Generalized Additive Models (GAMs). Specifically, a Negative Binomial distribution was applied for modeling q0, while a Gamma distribution with a logarithmic link function was used for q1 and q2. All models were fitted using the gam function from the mgcv package.

##### 2.4.2.2 β Diversity

We assessed beta diversity using Hellinger‐transformed abundance data with square‐root transformed dissimilarity matrices. To compare species homogeneity across habitat gradients, we calculated the total sum of squares (SS_total_) and the total beta diversity value (BD_total_), the species contribution to beta diversity (SCBD), the local contribution to beta diversity (LCBD), and the significance of LCBD indices (p.LCBD) using the beta.div function of the adespatial package (Dray et al. 2023). SCBD and LCBD represented the relative importance of each species and ecological uniqueness of each site to overall beta diversity in a region, respectively, with *p* values associated with the LCBD indices (Leão et al. 2020). A permutation test with 999 permutations is applied to evaluate the p.LCBD. The calculation method followed that of Legendre and De Cáceres (2013) and da Silva et al. (2018) (as follows):

$$S_{\mathrm{ij}}={\left(y_{\mathrm{ij}}-{\bar{y}}_{j}\right)}^{2}$$

$y_{\mathrm{ij}}$ denoted the individual value within the data matrix, where columns represent abundance values of n species across p sampling stations. The subscript i corresponds to sampling units, while j denotes species. $S_{\mathrm{ij}}$ is defined as the squared deviation of $y_{\mathrm{ij}}$ from the mean abundance of species j. In instances where all sampling sites exhibit equivalent abundance for a given species, the corresponding $S_{\mathrm{ij}}$ values in that column equal zero.

$${\mathrm{SS}}_{\text{total}}=\sum_{i=1}^{n}\sum_{j=1}^{p}S_{\mathrm{ij}}$$

The total sum of squares for the species composition data is denoted by ${\mathrm{SS}}_{\text{total}}$.

$${\mathrm{BD}}_{\text{total}}={\mathrm{SS}}_{\text{total}}/\left(n-1\right)$$

n is the number of species.

$${\mathrm{SS}}_{j}=\sum_{i=1}^{n}S_{\mathrm{ij}}$$

${\mathrm{SS}}_{j}$ represents the sum of squares for the species j, it is calculated to assess its contribution to overall beta diversity. A higher SCBD value indicates that a species contributes more to the overall beta diversity and has a more heterogeneous distribution across the study area.

$${\text{SCBD}}_{j}={\mathrm{SS}}_{j}/{\mathrm{SS}}_{\text{total}}$$

${\text{SCBD}}_{j}$ is the contribution of species j to beta diversity.

$${\mathrm{SS}}_{i}=\sum_{j=1}^{n}S_{\mathrm{ij}}$$

${\mathrm{SS}}_{i}$ is the uniqueness proportion of beta diversity in the sampling station i.

$${\text{LCBD}}_{i}={\mathrm{SS}}_{i}/{\mathrm{SS}}_{\text{total}}$$

${\text{LCBD}}_{i}$ is the contribution of site i to beta diversity. Higher LCBD values suggest that a site is more unique in its species composition compared to other sites.

We examined associations between LCBD values and physical habitat characteristics (substrate composition, river intervention status, and macrophyte cover) using a GAM with a Gamma distribution and logarithmic link function. Using simple linear regression models implemented with the lm function from the R stats package, we examined the relationships between: (1) species abundance and their SCBD values to explore whether rare species are more responsible for creating ecological uniqueness between sites, (2) site‐specific total abundance and LCBD values to understand if the site with higher abundance is more or less compositionally unique. Beta diversity calculations were conducted using the beta.div function (adespatial package; Dray et al. 2023).

Differences in abundance and biomass across habitat gradients were assessed using either one‐way ANOVA or Kruskal–Wallis tests. Abundance and biomass data were log (*x* + 1)‐transformed. We first tested for normality using the shapiro.test function. For variables that were normally distributed, we applied one‐way ANOVA, followed by Tukey's HSD test using the aov and PostHocTest functions. For variables that were not normally distributed, we used the Kruskal–Wallis test followed by Dunn's test, using the kruskal.test and kruskalmc functions. Simple linear regression models (lm function) were used to analyze the relationships of macrophyte coverage with abundance and biomass.

#### 2.4.3 Abundance‐Biomass Structure Variation

The Abundance Biomass Comparison (ABC curves) method was based on the theoretical background of evolutionary theory (R‐selection and K‐selection), used to assess the ecological status or detect evidence of disturbance in the fish community (Warwick 1986; Yemane et al. 2005). The *W* statistic quantifies the pattern observed in the ABC curves. Using the *W* statistic, we assessed the ecological status of fish assemblages across ecological gradients and potential anthropogenic influences at each site, which provides a basis for prioritizing streams and locations for conservation efforts. A positive *W*‐value indicates a stable community dominated by large‐bodied species; values near zero suggest a balanced or random distribution of biomass and abundance; and negative *W*‐values reflect a community characterized by high numerical abundance but low overall biomass. The *W* statistic was calculated for each site using the formula (Yang et al. 2021):

$$W=\sum \left(B_{i}-A_{i}\right)/\left(S-1\right)$$

where $B_{i}$ and $A_{i}$ are the cumulative proportions of biomass and abundance for species i, and *S* is the total number of species. We employed a Generalized Additive Mixed Model (GAMM) to examine how the *W* statistic related to physical habitat characteristics, including macrophyte cover, substrate type, and regulation, using the gam function with a Gaussian distribution and an identity link function. To account for natural longitudinal gradients along the stream, reach (sampling position) was included as a random effect.

## 3 Results

### 3.1 Environmental Variations

**FIGURE 2:**
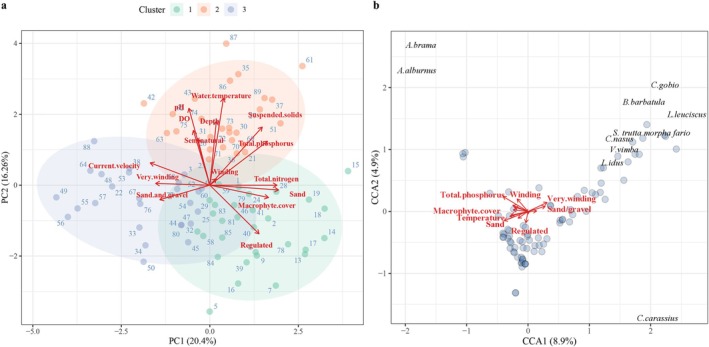
(a) Principal Component Analysis ordination of sampling sites based on morphohydrochemical habitat variables in the Drawa catchment, Pomerania (2010). Sites are grouped into three main clusters identified by *k*‐means clustering (*k* = 3), indicating separation in ordination space. Each numbered point corresponds to a sampling site. (b) Canonical Correspondence Analysis biplot illustrating the relationships among morphohydrochemical environmental variables, sampling sites, and fish assemblage species. The length and direction of the arrows indicate the relative influence and gradient direction of each environmental variable. Sampling sites are represented by blue circles. Only species with CCA scores greater than 1 or less than −1 are displayed, indicating their strong associations with the environmental variables.

Local morphohydrochemical conditions exhibited distinct spatial variability, as illustrated in reduced dimensional space and grouped by environmental similarity (Figure 2a). The first two axes of the PCA accounted for 36.66% of the total variation in habitat conditions. Component 1 characterized a gradient primarily influenced by substrate sand, total nitrogen, current velocity, macrophyte cover, and total phosphorus. Component 2 was mainly determined by water temperature, pH, depth, and suspended solids. Regulated river segments were generally associated with higher macrophyte cover and reduced current velocity. Sites exhibiting elevated suspended solids and pH often occurred on semi‐natural channels and had higher temperatures. Furthermore, highly sinuous (very winding) courses were strongly linked to lower flow velocities, and the stream temperature was highly correlated with depth, with deeper streams tending to be warmer.

### 3.2 The Role of Physical Habitat in Structuring Fish Communities

CCA axes 1 and 2 (CCA1 and CCA2) explained 8.9% and 4.9% of the variation in species–environment relationships, respectively (Figure 2b). A permutation test confirmed the overall model was statistically significant (*F* = 1.77, df = 15, *p* = 0.001). Temperature, total phosphorus, suspended solids, macrophyte cover, sand, sand/gravel, and very winding were primarily associated with CCA1, while regulated and winding were mainly associated with CCA2. Among the 15 environmental variables assessed, six significantly influenced fish assemblage structure (*p* < 0.05, Table 1). These included two chemical parameters (water temperature and total phosphorus), three physical factors (such as macrophyte cover, sandy substrate, and channel regulation), and the hydrological factor of stream depth. The results verified three key physical variables that significantly influenced fish assemblage structure: macrophyte coverage, substrate type, and river intervention status.

**TABLE 1:** Results of the Canonical Correspondence Analysis examining relationships among fish assemblage species, sampling sites, and morphohydrochemical environmental variables (with multicollinearity VIF < 4) in the Drawa catchment (2010).

Environmental variables	CCA1 (*p* = 0.001)	CCA2 (*p* < 0.05)	*F*‐Ratio	*p*
Water temperature	−0.486	−0.178	2.967	0.001***
DO	−0.264	−0.241	1.238	0.232
pH	−0.035	−0.041	1.012	0.399
Total nitrogen	−0.394	0.059	1.644	0.051
Total phosphorus	−0.665	0.534	4.268	0.001***
Suspended solids	−0.405	0.221	1.127	0.278
Current velocity	0.231	0.016	1.271	0.183
Macrophyte cover	−0.453	−0.134	1.815	0.034*
Depth	0.005	−0.087	1.706	0.042*
Sand	−0.635	−0.329	2.916	0.003**
Sand/gravel	0.459	0.183	1.114	0.322
Regulated	−0.057	−0.401	1.771	0.031*
Semi‐natural	−0.362	0.155	1.227	0.232
Very winding	0.509	0.323	1.143	0.281
Winding	−0.289	0.447	1.361	0.160

*Note:* The table includes CCA1 and CCA2 biplot scores for the environmental variables, along with significance testing results based on 999 permutations.

* Significant at *α* = 0.05.

** Significant at *α* = 0.01.

*** Significant at *α* = 0.001.

*V. vimba* , *C. gobio* , *L. idus* , *L. leuciscus* , *C. carassius* , *A. brama* , *S. trutta morpha fario*, *B. barbatula* , *C. nasus* , and *A. alburnus* were primarily associated with CCA1 (Table S2). With the exception of *C. nasus* , these species showed positive associations with highly meandering river sections and sand/gravel substrates, while exhibiting negative associations with macrophyte cover, water temperature, and sandy substrates. Additionally, *A. brama* and *A. alburnus* were also linked to CCA2, showing associations with total phosphorus concentrations and river sinuosity (winding).

### 3.3 Fish Composition, Abundance, and Biomass Along Habitat Gradients

**FIGURE 3:**
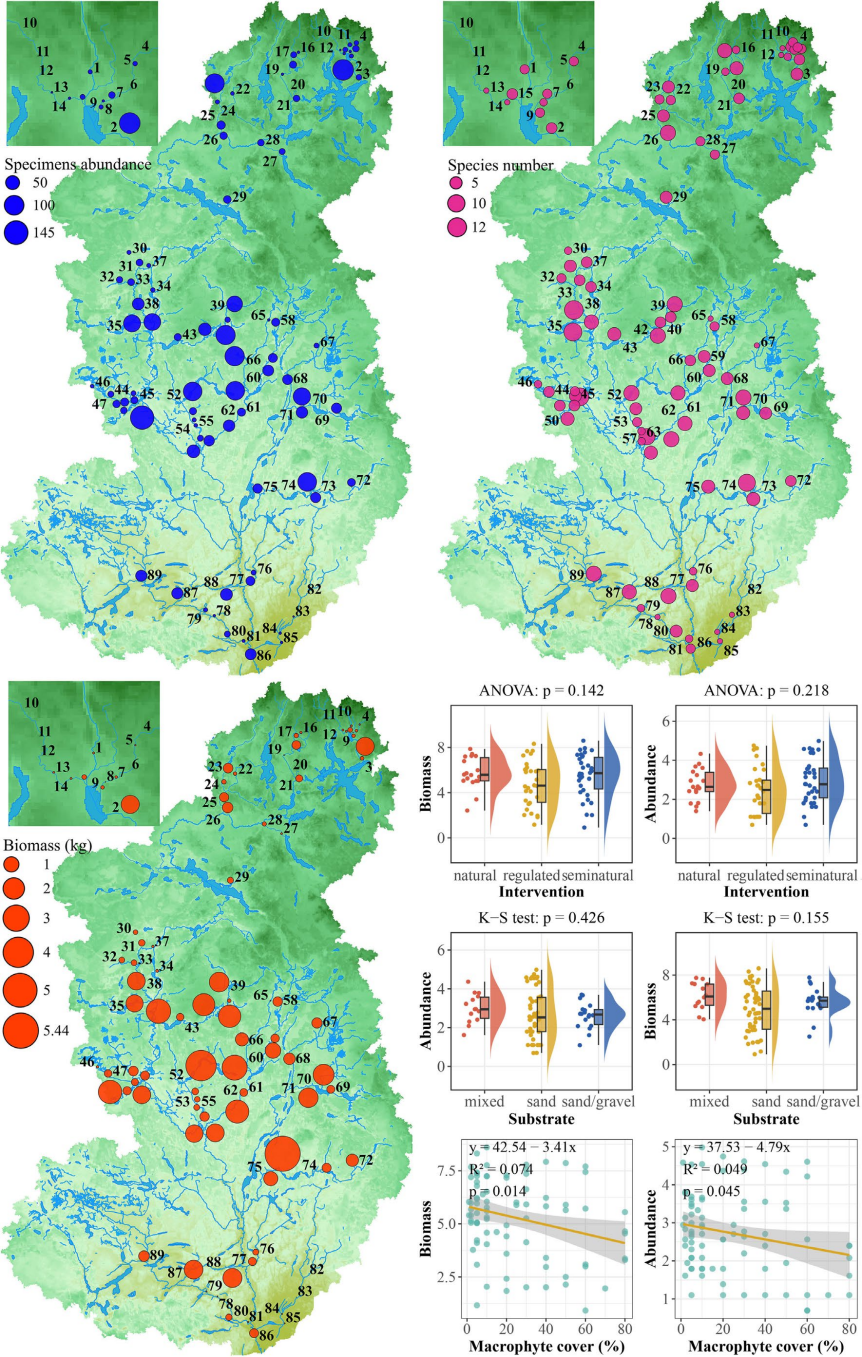
Specimen abundance, species number, and biomass distribution pattern (Drawa catchment, 2010). Variation in specimen abundance, species number, and biomass across habitat types was assessed using one‐way ANOVA or Kruskal–Wallis tests and linear regression model.

A total of 2136 individuals have been identified during the investigation, weighing 54.65 kg, classified into 29 species, 28 genera, 15 families, and 7 orders. In general, species number, specimen abundance, and biomass were highest in the middle reach. Specifically, the highest specimen abundance, biomass, and species number were recorded in site 51 (Prosta in middle reach), site 74 (Cieszynka in lower reach), and site 38 (Stara Drawa in middle reach), respectively (Figure 3, Table S1).

The species *Squalius cephalus, Salmo trutta morpha fario, Esox lucius, Rutilus rutilus, Perca fluviatilis, Gobio gobio, Anguilla anguilla, Lota lota, Alburnus alburnus*, and *Blicca bjoerkna* comprised 96.7% of the total biomass and 85.6% of the total abundance. *S. cephalus* was the dominant species by biomass, contributing 20.5% to the total fish assemblage biomass. *G. gobio* was the most abundant species, representing 19.9% of the total fish assemblage abundance (Table 2).

**TABLE 2:** Species composition in the Drawa catchment (2010).

Latin name	Abundance (%)	Biomass (%)
*Gobio gobio* L. 1758	19.94	6.78
*Rutilus rutilus* L. 1758	16.48	10.29
*Perca fluviatilis* L. 1758	14	10.28
*Squalius cephalus* L. 1758	8.29	20.53
*Blicca bjoerkna* L. 1758	7.16	3.61
*Salmo trutta morpha fario* L. 1758	6.51	17.21
*Alburnus alburnus* L. 1758	6.41	3.8
*Esox lucius* L. 1758	4.73	11.05
*Leuciscus leuciscus* L. 1758	2.81	1.19
*Cobitis taenia* L. 1758	2.06	0.29
*Tinca tinca* L. 1758	2.06	1.82
*Lota lota* L. 1758	1.92	4.47
*Cottus gobio* L. 1758	1.64	0.35
*Rhodeus sericeus amarus* Bloch 1782	1.17	0.07
*Gasterosteus aculeatus* L. 1758	0.98	0.06
*Misgurnus fossilis* L. 1758	0.84	0.34
*Scardinius erythrophthalmus* L. 1758	0.75	0.61
*Abramis brama* L. 1758	0.37	0.5
*Cyprinus carpio* L. 1758	0.37	0.17
*Phoxinus phoxinus* L. 1758	0.37	0.02
*Thymallus thymallus* L. 1758	0.28	0.54
*Leuciscus idus* L. 1758	0.19	0.1
*Anguilla anguilla* L. 1758	0.14	5.71
*Barbatula barbatula* L. 1758	0.14	0.05
*Chondrostoma nasus* L. 1758	0.09	0.03
*Vimba vimba* L. 1758	0.09	0.04
*Gymnocephalus cernuus* L. 1758	0.09	0.05
*Carassius carassius* L. 1758	0.05	0
*Lampetra planeri* L. 1758	0.05	0.03

Aquatic macrophytes significantly influence the biomass and abundance of the fish assemblage, whereas river regulation status and substrate type show no significant influence (Figure 3). Biomass (*F*(2, 79) = 2.00, *p* > 0.05) and abundance (*F*(2, 79) = 1.55, *p* > 0.05) did not show significant differences across the different river regulation levels. Similarly, no significant differences were observed for biomass (χ^2^ = 3.72, df = 2, *p* > 0.05) and abundance (χ^2^ = 1.71, df = 2, *p* > 0.05) among the substrate types. However, macrophyte cover was significantly associated with both biomass (*F*(1, 80) = 6.35, *p* < 0.05) and abundance (*F*(1, 80) = 4.14, *p* < 0.05).

### 3.4 Variations of Diversity Along the Habitat Gradients

**FIGURE 4:**
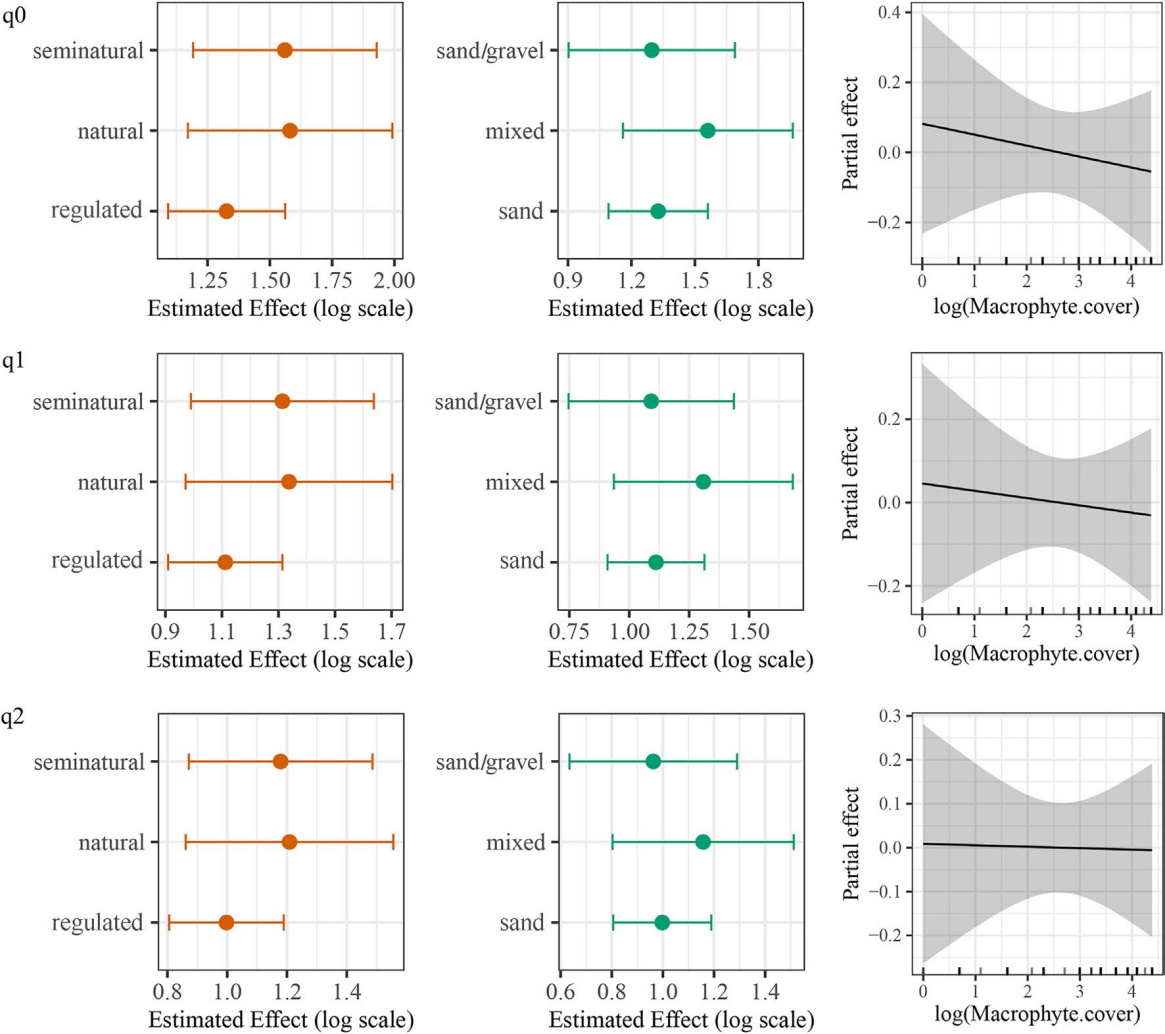
Patterns of α‐diversity (species richness: q0; exponential Shannon: q1; inverse Simpson: q2) across habitat gradients (intervention status, substrate type, and macrophyte cover) in the Drawa catchment (2010), as modeled by generalized additive models. No significant effects were found (*p* > 0.05). The figure presents fitted GAM estimates for intervention and substrate, and partial smooth effects with 95% confidence intervals for macrophyte cover.

Generalized Additive Models showed non‐significant patterns in Hill diversity indices (q0, q1, and q2) across habitat types. For species richness (q0), the model (adj. *R* ^2^ = 0.048; deviance explained = 10.1%) showed no significant effects of intervention (df = 2, χ^2^ = 3.18), substrate (df = 2, χ^2^ = 2.48), or macrophyte cover (edf = 1.0, χ^2^ = 0.30, all *p* > 0.05). For exponential Shannon (q1), the model (adj. *R* ^2^ = 0.026; deviance explained = 7.65%) also showed no significant effects of intervention (χ^2^ = 1.56), substrate (χ^2^ = 0.91), or macrophyte cover (edf = 1.0, *F* = 0.11; all *p* > 0.05). For inverse Simpson (q2), the model (adj. *R* ^2^ = 0.007; deviance explained = 6.19%) similarly showed no significant effects (intervention χ^2^ = 1.44, substrate χ^2^ = 0.73, macrophyte cover edf = 1.0, *F* = 0.004; *p* > 0.05; Figure 4).

**FIGURE 5:**
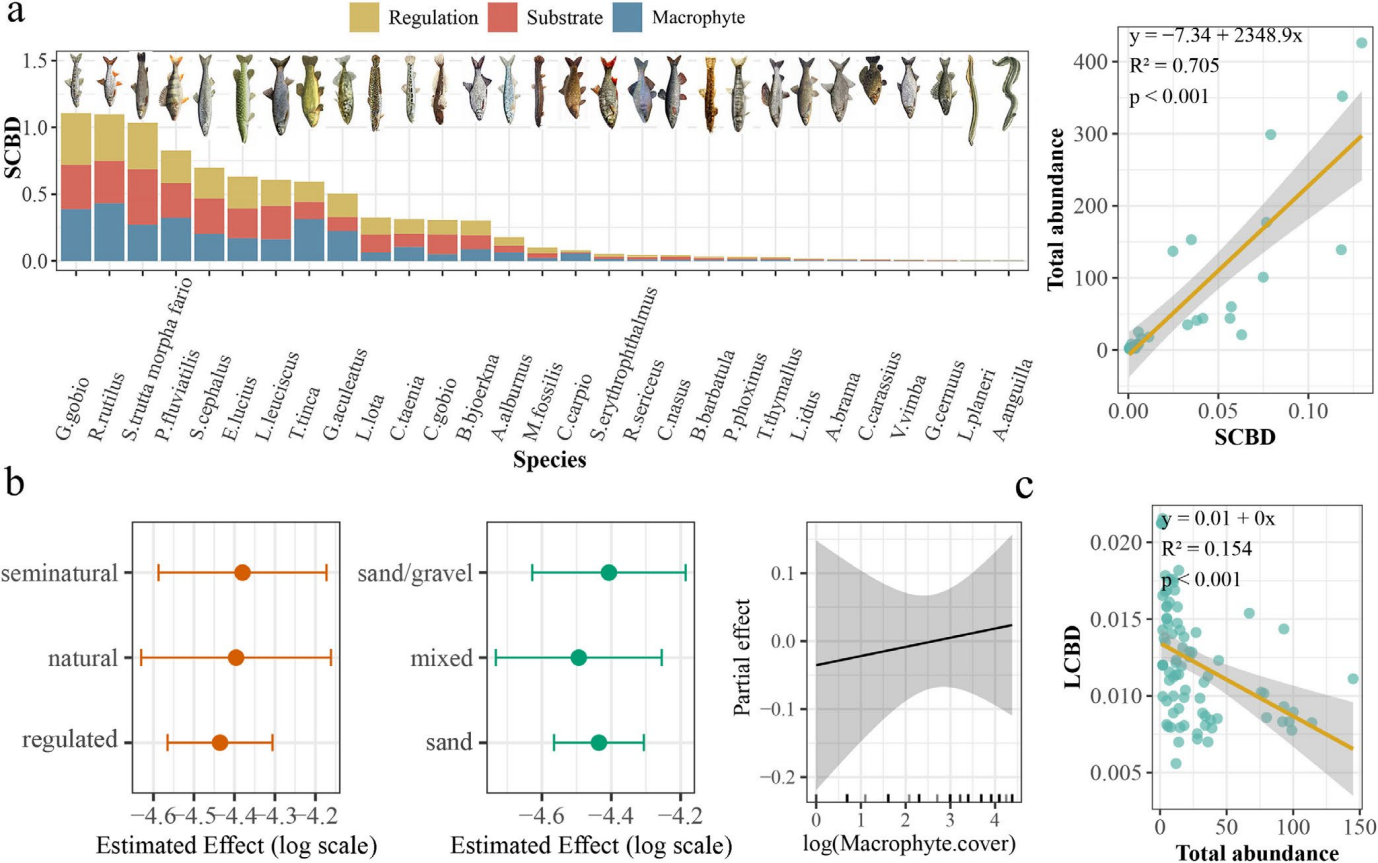
(a) Species Contributions to Beta Diversity (SCBD) under varying habitat conditions, including river regulation, substrate type, and macrophyte cover (left), and the relationship between SCBD and species abundance (right). (b) Local Contributions to Beta Diversity (LCBD) modeled along habitat gradients using GAMs. No significant effects were detected (*p* > 0.05). The plots show fitted GAM estimates for intervention and substrate type, and partial smooth effects of macrophyte cover with 95% confidence intervals. (c) Relationship between LCBD values and site‐specific species abundance in the Drawa catchment (2010).

*G. gobio* , *S. trutta morpha fario*, and *R. rutilus* made the highest contributions to beta diversity across both substrate type and river intervention status. For macrophyte, the highest SCBD values were observed in *G. gobio* , *R. rutilus* , *P. fluviatilis* , and *T. tinca* . Results showed a significant positive relationship between overall species' SCBD values and their abundance (*p* < 0.001, Figure 5a), suggesting that species with higher abundance play a greater role in structuring beta diversity.

Our results revealed that the total sum of squares and beta diversity values were 55.117 and 0.681 in the Drawa River catchment. This finding suggests a moderate level of species turnover, indicating some overlap in species composition between ecosystems within the catchment, while also highlighting the presence of species unique to each ecosystem. p.LCBD identified statistically significant sites contributing to overall beta diversity in the Drawa River catchment (sites 13, 14, 45, 46, 65, and 83). These sites had both relatively high LCBD values (0.021, 0.021, 0.018, 0.018, 0.022, and 0.021 respectively) and low *p*‐values (*p* < 0.05), indicating a strong contribution to variation in species composition across the catchment (Table S1 in Data S1).

Higher LCBD values reflect greater ecological distinctiveness. The Generalized Additive Model indicated no significant effects of substrate type, intervention status, or macrophyte cover on LCBD (adjusted *R* ^2^ = −0.05; deviance explained = 1.45%; Figure 5b). LCBD values were negatively correlated with site‐specific total abundance (*F*(1, 80) = 14.58, *R* ^2^ = 0.154, *p* < 0.001, Figure 5c), suggesting that sites with lower abundance are more compositionally unique.

### 3.5 Community Variation: Abundance‐Biomass Structure

**FIGURE 6:**
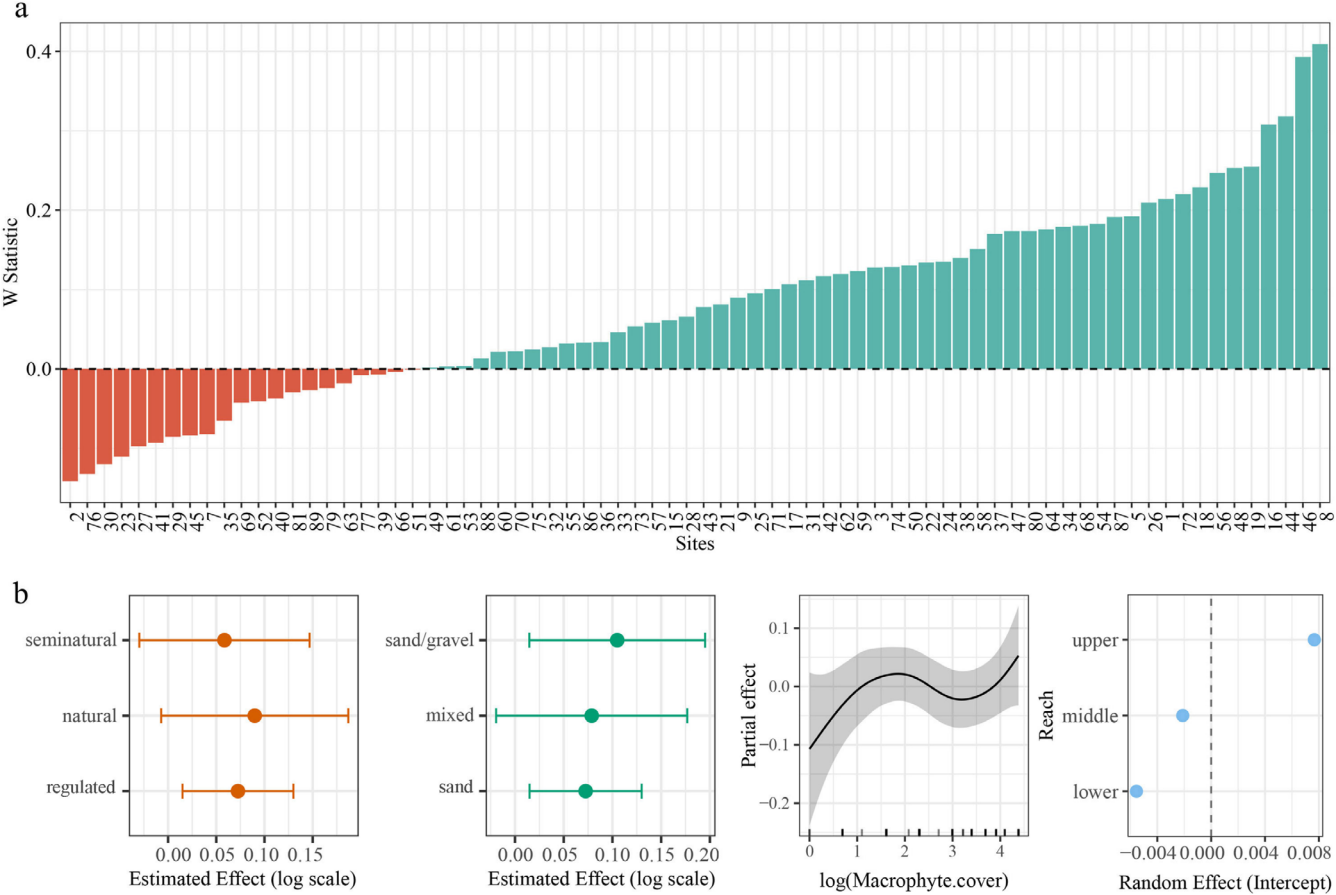
Generalized Additive Mixed Model results showing the estimated response of the W statistic to macrophyte cover, substrate type, and river intervention status in the Drawa catchment (2010). Plots display estimated effects with 95% confidence intervals. None of the fixed effects were statistically significant (*p* > 0.05). The random effect component was also not statistically significant (*p* > 0.05), indicating its variance did not significantly contribute to explaining variability in the *W* statistics.

Because fewer fish were recorded at some sites, *W* statistics could not be calculated for eight sites: site 13, site 14, site 65, site 67, site 78, site 83, site 84, and site 85. These sites, located in the upper, middle, and lower reaches of the catchment (Lipinka and Kamienna in the upper reach, Plociczna in the middle reach, and Pokretna and Czlopica in the lower reach), were excluded from the subsequent analysis and visualization. Based on W‐statistics analysis, 21 sites (site 2, site 7, site 23, site 27, site 29, site 30, site 35, site 39, site 40, site 41, site 45, site 51, site 52, site 63, site 66, site 69, site 76, site 77, site 79, site 81, and site 89) exhibited negative W values, indicating communities dominated numerically by small‐bodied organisms (Figure 6a). These sites are located on the Drawa headwater, Kokna, Wasowka, Stawna, and Prosna in the upper reach; the Radowka, Drawica, Bagnica, Stary potok, Slopica, Korytnica, Kamienna, and Prosta in the middle reach; and the Szczuczna, Mlynowka, Pokretna, and Koczynka in the lower reach. Additionally, 20 sites demonstrated W‐statistics values between 0 and 0.1, suggesting these communities may be dominated by smaller, opportunistic fish species (Figure 6a). These sites are located on the Lipinka, Wasowka, Miedznilk, Prosna, and Kokna in the upper reach; Sitna, Korytnica, Slopica, Pilknica, Stary potok, and Drawica in the middle reach; Mierzecka struga, Mlynowka, Cieszynka, and Czlopica in the lower reach.

The GAMM showed no significant effects of macrophyte cover (*F* = 1.40, edf = 3.37, *p* > 0.05), substrate type (*F* = 0.43, df = 2, *p* > 0.05), or river intervention status (*F* = 0.35, df = 2, *p* > 0.05) on *W*‐statistics. Reach contributed minimal variation (*F* = 0.26, edf = 0.44, *p* > 0.05; Figure 6b).

## 4 Discussion

Effective conservation of species may require a comprehensive understanding of large‐scale patterns in species richness and distribution (Liu et al. 2021). Our study investigated environmental variations of streams and the influence of physical habitat gradients on fish composition, diversity, and disturbance status. This information can be used to examine spatial patterns of fish assemblages, identify current and potential threats, and prioritize areas for reserve designation within the catchment.

### 4.1 Role of Environmental Conditions in Habitats

Human‐driven water stress, including dam construction, water abstraction, and channelization, affects the chemical composition of components within river ecosystems (Sabater et al. 2018). We investigated the presence of similar threats in the Drawa River catchment, where water chemical conditions are influenced by multiple interacting factors. While macrophytes can potentially improve water quality, their extensive growth in nutrient‐rich conditions can create problems such as impeding river flow (O'Brien et al. 2014). High macrophyte coverage was positively correlated with total nitrogen and regulated status, and negatively correlated with current velocity in our PCA analysis, suggesting dense macrophyte zones have slower water flow that limits oxygen exchange. We identified 12 sites with dissolved oxygen below 3 mg/L in our agricultural‐forested catchment, all having > 50% macrophyte cover. These oxygen‐depleted locations featured sandy beds and regulated channels, reducing turbulence, aeration, and filtration. Therefore, the unfavorable habitat conditions observed in the catchment result from the interaction of multiple factors rather than a single cause. This suggests that future water quality management strategies should adopt a comprehensive approach, addressing both site‐specific challenges and common factors affecting the entire catchment.

Ongoing macrophyte monitoring in the main channels and larger rivers suggests favorable ecological conditions within the study catchment (Domagała et al. 2015). Water chemistry, notably temperature and total phosphorus, significantly influences the structure of fish communities. This influence, and the variability of these parameters, is likely even more pronounced in smaller, less monitored streams and rivers. In these smaller water bodies, greater interaction with the surrounding environment leads to more pronounced fluctuations in water chemistry. Temperature and total phosphorus levels, for instance, can exhibit significant short‐term fluctuations (weekly, even daily) driven by factors such as wind‐wave energy, hydrodynamics, and key biological processes like phytoplankton biomass changes and algal blooms (Havens et al. 2007; Zhang et al. 2022; McIsaac et al. 2023). Stream depth is a crucial factor reflecting habitat size and is intrinsically linked to habitat diversity, complexity, and connectivity. Its variability is driven by the prevailing flow regime, air temperature, and precipitation patterns (Tsang et al. 2021). Studies have shown that streams experiencing greater hydrological variability, both spatially and temporally across years, exhibit lower assemblage stability and more pronounced fluctuations in organism density (Magoulick et al. 2021). Stream depth also serves as a significant factor influencing fish communities within the catchment. Consequently, the current focus on larger rivers leaves a critical monitoring gap in smaller tributaries, which serve as essential, sensitive habitats for larval and juvenile fish as well as migratory species and therefore require a high conservation priority.

Fish‐absent locations were identified in Prosna, Lipinka, Miedznilk, and Czlopica, all of which are human‐regulated channels with predominantly sandy substrates. These sites, except for Czlopica, are located in the upper reaches of the catchment and have poor habitat conditions for fish. Survey data from upper‐reach rivers where fish were absent indicated natural environmental conditions potentially limiting fish presence, including low primary productivity and characteristic headwater habitat features such as reduced current velocity and shallow depth. Critically, dissolved oxygen levels in these rivers were notably low (1.21–3.74 mg/L), a recognized limiting factor for fish abundance. For these rivers, further habitat investigations, effective water quality management, and channel reconfiguration should be prioritized. Czlopica in the lower reach presents a different case, with adequate oxygen levels (6.87 mg/L) but no fish presence. Given the lower water temperature during sampling (16.4°C), the absence of fish may be attributed to both lower species diversity in the location and fish preference for warmer locations.

### 4.2 Composition and Structure Variation Along Habitat Gradients

Approximately 80% of the fish species in this area are potamodromous, with a smaller proportion exhibiting anadromous (*n* = 4), catadromous (*n* = 1), and non‐migratory (*n* = 1) life histories. Most inhabiting species are long‐lived, exceeding 10 years, and primarily carnivorous. Our study found a greater number of fish species in the side channels compared to the lower mainstream of the Drawa River in 1994–1995 (Chelkowski et al. 1996). This difference was accompanied by species turnover, with six species disappearing and eight new ones colonizing the habitat.

According to the River Continuum Concept, stream order serves as a proxy for environmental conditions including substrate composition, river slope, and canopy cover (Strahler 1957; Vannote et al. 1980; Doretto et al. 2020). These physical attributes influence water temperature, chemistry, and light penetration, ultimately determining food resource availability in the ecosystem. However, natural discontinuities along a stream's longitudinal gradient in lowland riverine ecosystems, such as side channels and pools, can create variations in food resource availability and distribution (Doretto et al. 2020). In our study, the mean abundance, species richness, and biomass all peaked in the middle reach. This pattern may be attributed to higher environmental stress in the downstream sections of the catchment, such as a relatively higher number of both artificial and natural dams (Czerniawski and Bilski 2023).

Major environmental gradients, acting as abiotic factors, shape local assemblage structures (Tejerina‐Garro et al. 2005). Understanding these spatial patterns is critical for predicting community responses to environmental complexity (Moniruzzaman et al. 2021). CCA analysis revealed that macrophyte coverage, substrate type, and river intervention status significantly influenced fish community structure within the catchment. Rather than being shaped by individual factors, the fish community structure reflected the combined influence and interaction of multiple habitat variables. In general, straight and regulated channels were commonly associated with sandy substrates and varying macrophyte cover, whereas highly sinuous streams tended to have natural or less regulated characteristics and complex substrates. This pattern reflects the complex interactions among habitat factors throughout the catchment. River regulation affects various substrate types (including sand, sand‐gravel, and mixed substrates), with regulated rivers showing macrophyte coverage ranging from 2% to 80%. Despite the observed spatial homogeneity, habitat heterogeneity continues to exert an influence on fish community structure. Some species exhibit strong habitat preferences, while others are more generalist in their requirements (Szalóky et al. 2021). For instance, *C. gobio* , a small demersal fish, was entirely absent from sandy rivers. This positive correlation between its abundance and substrate complexity suggests a preference for habitats with gravel and rocky bottoms, aligning with previous findings (Legalle et al. 2005).

Our findings demonstrate substantial negative impacts of human activities, particularly river straightening, consistent with previous studies (Tejerina‐Garro et al. 2005; Grill et al. 2019) on fish communities. The dominance of *C. carassius* in regulated channels with higher total nitrogen and total phosphorus suggests a potential tolerance for these pollutants, an advantage in disturbed habitats (Kawamura 2008). Conversely, the absence of *A. brama* , a fish species known to utilize side channels during its juvenile stage for refuge from predation and resource acquisition (Skov et al. 2010; Slavík et al. 2024), underscores the importance of habitat complexity in the studied rivers. Similarly, *V. vimba* , an endangered anadromous fish, was exclusively found in natural channels free of dams, underlining the detrimental effects of water obstructions on migratory species. Widespread straightening (25 out of 27 channels) likely alters water flow patterns, reduces habitat heterogeneity, and potentially affects substrate composition, leading to a decline in fish species diversity and abundance.

Vegetated areas typically host more fish than bare habitats, with moderate macrophyte density providing optimal conditions for fish diversity, growth, and survival (Schultz and Dibble 2012). However, our results revealed a significant negative correlation between macrophyte coverage and fish populations, with both fish biomass and abundance decreasing as macrophyte density increased in the streams. This observed pattern, where areas with denser aquatic macrophyte supported lower fish abundance and biomass compared to those with sparser macrophyte, may reflect either an inhibitory effect of dense macrophyte on fish populations or a diminished sampling efficiency of wading electrofishing in densely vegetated reaches, potentially leading to an underestimation of the actual fish community. The variability in fish size and feeding behavior may stem from different species' distinct responses to vegetated habitats, depending on their ecological requirements. For example, *S. trutta morpha fario* was far more abundant in sparsely vegetated habitats with macrophytes (122 specimens) compared to only three specimens found in densely vegetated habitats with macrophytes. On the other hand, excessive macrophyte growth creates unfavorable water conditions in the catchments and may be further complicated by potential exotic invasive plants displacing native species, resulting in simplified plant communities that provide poorer food resources for macroinvertebrates and fish (Schultz and Dibble 2012; Havel et al. 2015).

### 4.3 Fish Diversity Patterns

Using Hill numbers, where q0 represents total species count, q1 weighs species by their frequencies, and q2 gives more weight to common species, we analyzed community diversity patterns (Jost 2006). We found that substrate type, human intervention status, and macrophyte coverage had no significant effect on Hill numbers. Across habitat gradients, the contribution of individual species to beta diversity varies. This variation can serve as a valuable tool for managers to identify key species that might be sensitive to physical changes or human interventions in the area, as these factors could influence their distribution or abundance. For instance, *G. gobio* , a widespread and abundant species during the study, contributed significantly to the observed differences between habitat types.

The SCBD value aids in understanding a species' relative influence on the overall variation in a community's species composition. Specialists with narrow niches tend to have higher SCBD values compared to generalist species. This is because specialists are confined to specific environmental conditions, and their presence or absence significantly influences the overall community composition. Species with an intermediate occupancy rate (neither rare nor widespread) often have the highest SCBD values (Heino and Grönroos 2017). A strong positive correlation between species abundance and SCBD values was observed. Our study identified *G. gobio* , *R. rutilus* , and *S. trutta morpha fario* as the species with the highest SCBD values within the catchment. While *S. trutta morpha fario* exhibited the highest mean abundance in rivers with natural channels and mixed substrates, *R. rutilus* and *G. gobio* were more abundant in semi‐natural rivers. On the other hand, *S. trutta morpha fario* preferred habitats with sparse macrophyte, whereas *R. rutilus* and *G. gobio* were more abundant in areas with moderate macrophyte. The high SCBD values for these species underscore their distinct habitat preferences, particularly *S. trutta morpha fario*, which appears to favor more complex physical environments with less macrophyte.

LCBD values displayed different responses to habitat gradients. Sites with the lowest LCBD values (18, 50, and 63) also harbored the most species (6, 6, and 8 species, respectively). These reaches exhibit a diverse range of physical habitat conditions, suggesting that these sites possess good ecological health, regardless of their habitat complexity and level of human intervention. Despite their lower LCBD values, indicating less unique biodiversity, these sites could be potential candidates for ecological restoration efforts (Legendre and De Cáceres 2013). Sites 13, 14, 65, and 83, which were characterized by low species richness (each site harboring only one species: *T. tinca* , *C. taenia* , or *G. aculeatus* ), were particularly notable. Sites with higher LCBD values generally had lower species abundance. These high LCBD values suggest that these sites harbor relatively unique species assemblages and may contain special habitat types or specific environmental conditions. Consequently, they can be considered prime candidates for conservation prioritization (Legendre and De Cáceres 2013).

### 4.4 Ecological Characteristics of Biomass‐Abundance Structure

The W‐statistic provides a quantitative measure of community status. By analyzing patterns in abundance and biomass distributions, this index can be used to assess environmental condition (Warwick 1986; Yemane et al. 2005; Di Lorenzo et al. 2022). It is a sensitive indicator that supports ecosystem‐based bioassessment, but due to limitations in naturally species‐poor ecosystems, it should be integrated with other assessment frameworks for effective evaluation (Marín‐Guirao et al. 2005; Stojković Piperac et al. 2016). While habitat characteristics did not significantly influence the *W*‐statistics, the computation of *W*‐statistics could not be performed at several sites due to low specimen abundance. It is noteworthy that these particular sites predominantly comprised regulated or semi‐natural rivers, with seven out of eight of them distinguished by sandy substrates.

In general, over half of the surveyed sites exhibited *W*‐statistics ranging from −0.2 to 0.1, indicating a predominance of smaller bodied fish species within these communities. Notably, many rivers associated with lower *W*‐statistics contain anthropogenic structures such as culverts, floodgates, road crossings, and hydropower stations of various scales. The artificial barriers, built on small rivers, significantly impact fish assemblages through habitat alterations (Moreira et al. 2023).

The findings indicate that, while fish communities throughout the catchment are composed of individuals with generally smaller body sizes, the impact is more severe in habitats with simpler physical structure and highly anthropogenic influence. The results derived from the analysis of the *W*‐statistics were in concordance with other components of the study, thereby supporting the overall ecological interpretations.

## 5 Conclusion

The study found that habitat diversity, mediated by physical environmental differences, significantly impacted the community structure of small stream fish and their disturbed status. Firstly, the PCA results reveal distinct spatial variability in local morphohydrochemical conditions, highlighting the influence of multiple environmental factors on species distribution and underscoring the need for comprehensive aquatic monitoring. Effective water quality management is particularly urgent at sites where fish are absent. Secondly, physical environmental gradients within the Drawa catchment significantly influence the structure of fish assemblages. Streams with less dense macrophyte cover significantly showed higher fish abundance and biomass in the catchment. Species exhibiting higher relative abundance tend to exert a stronger influence on shaping patterns of beta diversity, whereas sites characterized by lower overall abundance often display greater compositional uniqueness in their fish communities. Finally, fish communities in natural habitats exhibited larger body sizes compared to those in simplified habitats (those with sandy or muddy substrates, channelization, and sparse macrophyte). High local fish biodiversity, interconnected habitat patches, and high habitat heterogeneity can promote the resilience of river fish populations against flood pulses, global warming, and other environmental changes (Stoffers et al. 2022). Therefore, this study may contribute to more effective habitat restoration and conservation of fish assemblages. Targeting instream habitat improvement through increased substrate complexity, obstruction removal, fish passage construction, and channel reconfiguration in the Drawa catchment could be crucial for coping with threats attributed to human activities and global warming.

## Author Contributions

**Xiaohao Shi:** conceptualization (lead), methodology (lead), software (lead), visualization (lead), writing – original draft (equal). **Robert Czerniawski:** funding acquisition (lead), supervision (lead), writing – review and editing (equal).

## Conflicts of Interest

The authors declare no conflicts of interest.

## Supporting information


**Data S1:** ece372092‐sup‐0001‐DataS1.zip.
**Figure S1:** Representative examples of characteristic physical habitat types within the Drawa catchment. All photographs were taken by the research team and correspond to the standardized physical habitat classification scheme utilized in this study.

## Data Availability

The data that supports the findings of this study are available in the Data S1 of this article.
