# Supplementary figures and images for: Habitat Heterogeneity and Channelization of Small Rivers and Streams: Fish Community Responses to Ecological Gradients
Source: Ecol Evol. 2025 Sep 5;15(9):e72092. doi: 10.1002/ece3.72092 (PMC12413487; doi:10.1002/ece3.72092)

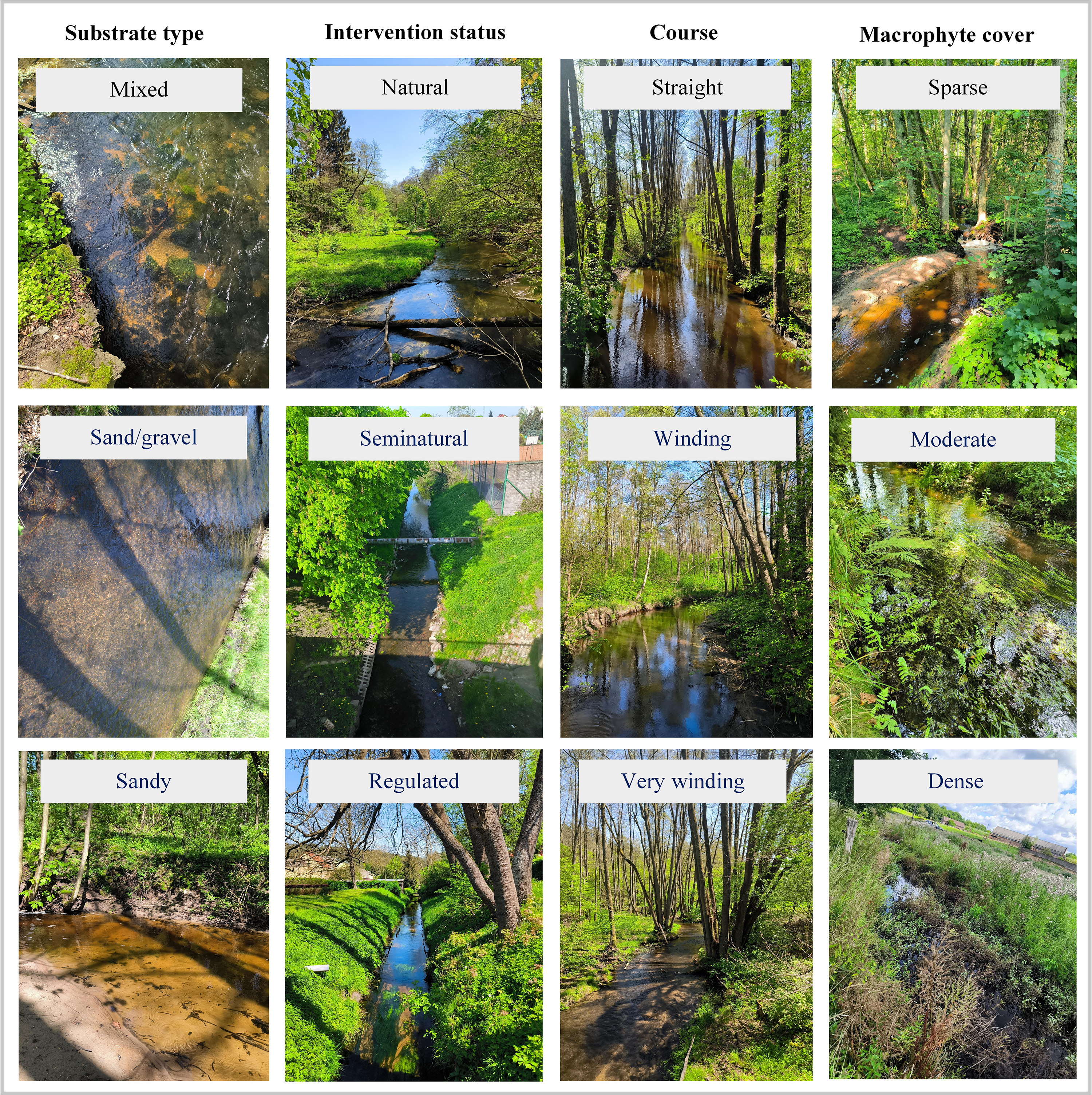

Supplement: Supplementary file 1 — Data S1: ece372092‐sup‐0001‐DataS1.zip. Figure S1: Representative examples of characteristic physical habitat types within the Drawa catchment. All photographs were taken by the research team and correspond to the standardized physical habitat classification scheme utilized in this study. [file ECE3-15-e72092-s001.zip › ece372092-sup-0003-FigureS1.png]
